# Cyberinfrastructure to Improve Forest Health and Productivity: The Role of Tree Databases in Connecting Genomes, Phenomes, and the Environment

**DOI:** 10.3389/fpls.2019.00813

**Published:** 2019-06-25

**Authors:** Jill L. Wegrzyn, Margaret A. Staton, Nathaniel R. Street, Dorrie Main, Emily Grau, Nic Herndon, Sean Buehler, Taylor Falk, Sumaira Zaman, Risharde Ramnath, Peter Richter, Lang Sun, Bradford Condon, Abdullah Almsaeed, Ming Chen, Chanaka Mannapperuma, Sook Jung, Stephen Ficklin

**Affiliations:** ^1^Department of Ecology and Evolutionary Biology, University of Connecticut, Storrs, CT, United States; ^2^Department of Entomology and Plant Pathology, University of Tennessee, Knoxville, Knoxville, TN, United States; ^3^Umeå Plant Science Centre, Department of Plant Physiology, Umeå University, Umeå, Sweden; ^4^Department of Horticulture, Washington State University, Pullman, WA, United States

**Keywords:** database, content management system, forest tree, bioinformatics, web services

## Abstract

Despite tremendous advancements in high throughput sequencing, the vast majority of tree genomes, and in particular, forest trees, remain elusive. Although primary databases store genetic resources for just over 2,000 forest tree species, these are largely focused on sequence storage, basic genome assemblies, and functional assignment through existing pipelines. The tree databases reviewed here serve as secondary repositories for community data. They vary in their focal species, the data they curate, and the analytics provided, but they are united in moving toward a goal of centralizing both data access and analysis. They provide frameworks to view and update annotations for complex genomes, interrogate systems level expression profiles, curate data for comparative genomics, and perform real-time analysis with genotype and phenotype data. The organism databases of today are no longer simply catalogs or containers of genetic information. These repositories represent integrated cyberinfrastructure that support cross-site queries and analysis in web-based environments. These resources are striving to integrate across diverse experimental designs, sequence types, and related measures through ontologies, community standards, and web services. Efficient, simple, and robust platforms that enhance the data generated by the research community, contribute to improving forest health and productivity.

## Introduction

Starting in the Sanger sequencing era, significant investments were made to catalog genetic resources in primary repositories (Frishman et al., 1998). EMBL (the European Molecular Biology Laboratory), DDBJ (the DNA Data Bank of Japan), and NCBI (the National Center for Biotechnology Information) GenBank were initiated between 1980 and 1992, and remain freely accessible and federally funded (Benson et al., 1997; Tateno and Gojobori, 1997). The vast majority of data for these large, sequence-centric databases is sourced directly from researcher submissions that are encouraged through peer review journals. These primary resources have evolved with the data collection and curation needs of today, expanding in terms of both the sequence source and the associated metadata (Sayers et al., 2019). All three specialize in generating persistent identifiers to track a single sequence over an extensive network of resources. A genic identifier, as an example, may link a reference genome in NCBI’s Genome, an expression value in the Gene Expression Omnibus (GEO), and support for a UniRef90 cluster. These uniquely accessioned resources are increasingly integrated into secondary and tertiary repositories that subset or enhance these accessions with data specific to the communities they serve (Herrero et al., 2016).

As the data types and experimental designs contributing to these repositories diversified, a plethora of model organism databases (MODs) or clade organism databases (CODs) emerged. These databases sought to provide unique resources for the research communities they serve, through layered curation and specialized integration. The AAtDB (An *Arabidopsis thaliana* Database), developed in 1991 to support the first model plant system, has since evolved into the widely accessed, Arabidopsis Information Resource (TAIR) (Flanders et al., 1998; Huala et al., 2001). Around the same time, USDA-ARS funds were dedicated to developing some of the first informatic portals for economically important crop species, including RiceGenes (Cartinhour, 1997), GrainGenes (Triticeae) (Carollo et al., 2005), MaizeGDB (Lawrence et al., 2004), SoyBase (Grant et al., 2009), and the Dendrome Project for forest trees (Wegrzyn et al., 2008). Some of these databases remain independent funded entities, while others have merged into larger repositories or broadened their scope. There are hundreds of plant-focused organismal databases acting as secondary repositories today (Lai et al., 2012; Chen et al., 2018). The vast majority have moved beyond genetics and genomics data, providing advanced integration through stock centers, phenotypic evaluation, breeding resources, and metabolomic pathway integration.

Forest trees are unique among species represented in crop databases. The vast majority are long-lived, outcrossing, with extensive natural distributions represented by large, diverse and locally adapted populations (Holliday et al., 2017). They represent species of economic importance and are used for paper, pulp, biofuels, food, and timber production. At the same time, they serve as a foundation for watersheds, biodiversity, and contribute substantially to carbon sequestration with forests covering roughly 30% of the earth’s surface (Houghton, 2005). Like many plants, forest trees have complex genomes with challenges associated with ploidy and repetitive content. Additionally, gymnosperm tree genomes are exceptionally large, ranging from 10 to 40 Gbp in size (De La Torre et al., 2014). This, in combination with the need to broadly sample these large and diverse populations, yields limited full genome representation. Of the nearly 60,000 forest tree species, less than 35 are associated with an assembled and annotated reference genome (Neale et al., 2013; Plomion et al., 2016). A view into our primary databases reveal that over 2,000 species are associated with genetic information that is of value to the research community (Sayers et al., 2019).

**FIGURE 1:**
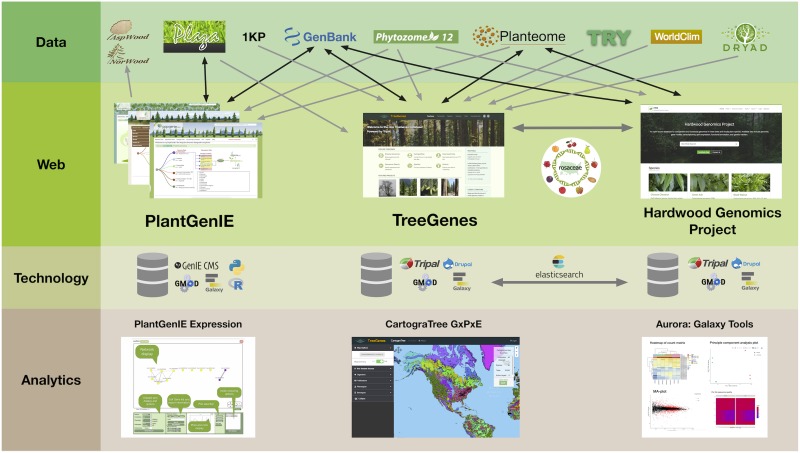
PlantGenIE, TreeGenes, and Hardwood Genomics Project represent integrated web-based frameworks that rely on a combination of primary repositories, secondary plant comparative databases, and user submissions to provide further value through data curation, integration, and analytics.

In the era of high throughput technologies that assess genotype, phenotype, and environmental metrics much faster than we can conceive, the need for well structured, efficient, and well-connected databases is apparent. In a recent survey of over 700 investigators in the biological sciences, access to analytical frameworks, long-term storage, and the ability to integrate data across disparate sources, were of primary concern (Barone et al., 2017). The long-lived nature of trees, their pivotal role in local economies, and role in ecosystem health requires an integrated approach that must leverage datasets from a variety of sources. To meet the needs of the research community, forest tree databases are moving away from independent database structures and toward integrated Content Management Systems (CMS) that support specific, shareable modules for query and analysis (Ficklin et al., 2011; Sundell et al., 2015). There is less focus on standardized database backends since web services allow users to expose their data, and data definitions. The ability to provide this, and cross-site query, relies heavily on the curation of ontologies to describe data housed in these frameworks. Initiatives that curate woody plant ontologies, to describe plant structures and measured traits, are critical components of forest tree database cross-talk (Jaiswal et al., 2005; Lens et al., 2012; Cooper et al., 2018). When these terms are provided within the framework of recommended standards for data collection, such as the Minimal Information About a Plant Phenotyping Experiment (MIAPPE), opportunities for analytical pipelines to evaluate complex data becomes a reality (Krajewski et al., 2015). In addition to these standards, all tree databases are integrated with existing analytical frameworks, such as Galaxy, that support and expose common bioinformatic workflows (Afgan et al., 2018). In this review, four tree databases are described, including their history, scope, current resources, and analytic tools (Figure 1).

## TreeGenes

TreeGenes^1^, previously known as Dendrome, was initially constructed to provide access to genetic data for forest trees in a relational framework. Early development included curation of molecular markers, genetic maps, Expressed Sequence Tags (ESTs), and species range maps. TreeGenes remained in a custom database schema and website, adopting components of the Generic Model Organism (GMOD) framework for housing genetic maps (cMAP) and genome assemblies (JBrowse) (Stein et al., 2002). Later development focused on the integration of genotyping resources, phenotypes, expression studies, and additional reference genome sequences (Wegrzyn et al., 2012; Falk et al., 2018).

TreeGenes currently represents just over 1700 species from 16 orders and 124 genera (Falk et al., 2018). TreeGenes has 1200 registered users with associated colleague accounts that enable access to data submission and analytical pipelines. The database contains 27 reference genomes, 100 genetic maps, 36.7 M genotypes, 303 species with transcriptomes, 40 species with TreeGenes’ Unigenes, 306 unique phenotype measures and 935,596 phenotypic measures. Genomic data is sourced primarily from GenBank, 1KP, Phytozome, and PLAZA (Goodstein et al., 2012; Matasci et al., 2014; Proost et al., 2015; Sayers et al., 2019). Phenotypic data is integrated from TRY-DB and Dryad, but primarily comes from direct user submissions (Kattge et al., 2011). Environmental data is extracted from imported layers, including temperature, precipitation, and solar radiation from WorldClim (Fick and Hijmans, 2017), and a variety of metrics from the Harmonized World Soil Survey Database (FAO et al., 2009).

TreeGenes is running on Tripal v3 which integrates a content management system known as Drupal with the Genetic Model Organism Database’s (GMOD) relational schema, known as Chado. This conversation in 2017, aligned TreeGenes for the first time with over 30, primarily plant, databases (Ficklin et al., 2011; Sanderson et al., 2013). Recent focused development in Tripal, led by the tree and legume community, enabled cross-site communication, access to efficient data transfer, and the ability to interface with a local installation of Galaxy (Watts and Feltus, 2017; Wytko et al., 2017). Galaxy is an independent framework that provides an API to abstract command line informatic software, develop workflows, and connect to high performance computing resources. Conversion into Tripal resulted in a complete overhaul of the database, and has enabled the development of several analytical modules that allow researchers to search, filter, and funnel data directly into supported workflows.

Following conversion, TreeGenes released a set of Tripal modules that can be utilized by researchers visiting the site or installed and customized for any Tripal supported databases. Tripal Sequence Similarity Search (TSeq) provides access to genomes, transcriptomes, and curated TreeGenes unigenes through traditional NCBI BLAST or optimized Diamond protein searches (Boratyn et al., 2013; Buchfink et al., 2015). The Tripal Plant PopGen Submit (TPPS) module presents a framework for researchers to submit their association genetics, landscape genomics, and related population studies by collecting any combination of molecular markers, phenotypes, and environmental measures. This module implements MIAPPE standards and the associated ontologies to ensure data integrity. The Tripal OrthoQuery module provides a framework for curating unigenes, executing OrthoFinder (Emms and Kelly, 2015), and generating interactive visualizations of gene families in a phylogenetic context. OrthoQuery enables both real-time orthologous gene family analysis and functional assessment of the resulting orthogroups.

Current development in TreeGenes focuses on CartograTree, which enables integration of genotype, phenotype and environmental data for georeferenced forest trees (Herndon et al., 2018). This module provides a robust framework to query publication datasets, species, phenotypes, genotypes, and associations based on metadata collected at the time of submission. The data and metadata exposed in CartograTree is derived from published population level studies submitted to TreeGenes via TPPS or curated from Dryad. Landscape genomics, association genetics, and population structure analysis is executed through the Galaxy framework.

## Hardwood Genomics Project

The Hardwood Genomics Project (HWG) provides access to genomic resources generated from angiosperm trees, including forest and urban trees of ecological and agricultural significance. The resource originated from the Fagaceae Genomics Web, built in 2007, to house transcriptomes, genomes and genetic maps. As new collaborators joined the effort and the scope of species extended beyond Fagaceae, the site was rebuilt in 2011 as the HWG. HWG’s mission is to host reference genomes and transcriptomes that are either not accessible elsewhere, or only available as raw files without an associated and searchable, functional annotation. In addition, HWG accepts molecular markers, genetic maps, germplasm and population descriptions, and community project descriptions. Current resources support species associated with pest or pathogen threats, including green ash, European ash, American chestnut, American beech, black walnut, and redbay, as well as trees with significant economic value, including white oak, black cherry, sugar maple, and tulip poplar.

For species with an available reference genome, HWG provides a workspace for accessing the annotation. This provides value to these sequence resources by performing and hosting functional annotation, including: identification of Open Reading Frames (ORFs) from transcripts, BLAST annotations derived from the Uniprot Swiss-Prot/TrEMBL plant protein databases, InterProScan domain searches (Jones et al., 2014), Gene Ontology (GO) term assignments (Ashburner et al., 2000), and predictions for Simple Sequence Repeats (SSRs) and primers. Researchers can download flat files, explore the spatial context of the assembly with JBrowse (Buels et al., 2016), search functional annotation for genes, and explore assigned GO terms through the ontology graphs. Additional genome specific data, such as alternative splicing, variants, and molecular markers are added to the JBrowse viewer when available.

Hardwood Genomics Project is currently running Tripal v3, and like TreeGenes, is responsible for the development of custom modules that can be installed on other Tripal-enabled sites. RNASeq data is poorly integrated in plant community databases despite the widespread use of expression studies to examine responses to biotic and abiotic stressors in plant systems. To address this limitation, HWG launched a framework devoted to the integration and analysis of gene expression experiments. BioSamples imported from GenBank, with the metadata describing the tissues, treatments, experimental design, and informatic methods, can be explored and compared. Each transcript, examined as part of an RNASeq experiment, has expression values that can be interrogated through interactive visualizations or downloaded for further analysis. The expression data displays can be customized interactively, grouping BioSamples by their tagged metadata values. A tool for comparing gene expression is also available, allowing the user to provide their own gene list and generate a heat map comparing expression of those genes across the relevant BioSamples (Chen et al., 2017). Current development in HWG is focused on supporting bioinformatic workflows, through Galaxy, to allow users to load their own datasets for analysis. HWG has also developed an Elastic Search module that enables search engine style cross-site query. This enables the discovery of relevant datasets within and across Tripal-enabled websites. The Aurora Galaxy Tripal module allows informatic tools to be wrapped in R Markdown which makes it possible to generate Galaxy workflow outputs as static websites.

## Genome Database for Rosaceae

The Genome Database for Rosaceae (GDR^2^, Jung et al., 2013) was initiated in 2003 to provide curated and integrated, genomic, genetics and breeding (GGB) data alongside analysis tools to enable basic, translational and applied research. Rosaceae is an economically, nutritionally and biologically important plant family that includes the majority of tree fruits (apple, apricot, blackberry, cherry, peach, plum), nuts (almond), and ornamentals (pear, crab apple). While not specifically focused on forest trees, GDR is included here for its role in developing Tripal modules for breeding and the comparative genomics utility with forest hardwoods.

GDR contains 21 genome assemblies and annotations for 14 species. A total of 528,890 genes, reference transcriptomes for all major species, 14,411 germplasm records, 313 genetic maps, 3.3 M molecular markers, 402,559 phenotype measurements, 3,902 QTL/MTL for 392 agronomic traits, 10.8 M genotypes, and 7,449 publications are housed in the database. GDR provides access to breeding management and analysis tools, pathway analysis through PlantCyc and Pathway Inspector, flexible front-end querying, genome annotations through JBrowse, and sequence similarity search functionality (Jung et al., 2016, 2017). GDR is participating in the development of new Tripal modules; visualization and analysis of genetic maps is available through the new Tripal Map Viewer module while whole genome alignments can be executed through the Tripal Synteny Viewer (Jung et al., 2018). GDR is currently expanding the analytic capabilities of their Breeding Information Management System (BIMS) and developing reference genome integration for the Tripal Map Viewer module.

## Plant Genome Integrative Explorer

The Plant Genome Integrative Explorer (PlantGenIE) began as The Populus Integrative Genome Explorer (PopGenIE; Sjödin et al., 2009), to overcome a lack of tools for routine tasks such as annotating gene lists, converting among sequence identifiers, and visualizing transcript abundance on the basis of EST sequencing. The Populus version was expanded to include visualization of poplar microarray data using the concept of the *Arabidopsis* electronic fluorescent pictograph (eFP) browser (Winter et al., 2007), gene set enrichment tests for Gene Ontology (Ashburner et al., 2000), Pfam (Finn et al., 2010), genome synteny browsing alongside sequence similarity searching. Later, a complimentary comparative co-expression tool was developed to facilitate inference of functional orthologs on the basis of conserved co-expression (Netotea et al., 2014). The resulting networks were integrated within *Populus* and *Arabidopsis* GenIE sites. With the release of the Norway spruce (*Picea abies*) genome (Nystedt et al., 2013), the associated resources were made available in a Conifer database, ConGenIE, which also includes genomes for loblolly pine (*Pinus taeda*; Neale et al., 2014; Wegrzyn et al., 2014) and white spruce (*Picea glauca*; Birol et al., 2013).

The PlantGenIE umbrella resource (Sundell et al., 2015), which included the development of new and updated gene expression tools, together with an integrated gene family analysis, is now available for all species. The primary aim is visualization of gene expression data, primarily from forest tree species, but including related sites for plant models, such as *Arabidopsis*. As such, gene expression resources for aspen (AspWood; Sundell et al., 2017) and Norway spruce (NorWood; Jokipii-Lukkari et al., 2017) cryogenic tangential cuttings series profiling wood development are being integrated within the PopGenIE and ConGenIE sites, respectively. Dedicated sites have been developed to provide access to spatial transcriptomics (Giacomello et al., 2017) and laser capture microdissection (Canas et al., 2014) gene expression data. For a subset of species, community annotation is also provided via WebApollo (Stevens et al., 2016).

The GenIE sites were originally developed using the Drupal CMS with many of the tools from GMOD (Stein et al., 2002). More recently, an alternative open-source CMS has been developed (GenIE-CMS^3^) and the PlantGenIE resource is currently being updated in this platform. GenIE-CMS includes web services, enabling end users to access genomic information from external interfaces such as R and Python analysis scripts. Alongside this update, new and improved versions of gene expression visualization tools have been developed and made available as plugins to GenIE-CMS. The PlantGenIE update includes integration with the PLAZA resource (Proost et al., 2015), integration of cross-GenIE gene lists using PLAZA gene orthology inference methods, new integrative gene expression explorer tools, new and updated gene expression networks inferred using seidr (Schiffthaler et al., 2018), and an updated functional enrichment tool. In addition to updating existing reference genomes, new genomes are being added, including a dedicated eucalyptus site, EucGenIE. The development of GenIE-CMS enables rapid and easy implementation of new GenIE resources and cross-linking among existing GenIE sites using PLAZA gene family and orthology information.

## Future Directions

Tree database cyberinfrastructure is supporting comparative genomics, population genetics, expression profiling, and genome annotation. These resources focus on a combination of model and non-model systems and integrate with established comparative resources to deliver value added information. Despite their importance, the sustainability of cyberinfrastructure and the related activities of curating and importing scientific data is always in question. The databases described here are leveraging larger open-source projects as their base framework and sharing web-based applications for common functionality, such as genome browsing and sequence similarity searching. For the forest tree community, the majority of the functionality described here has been deployed within the last 3 years and represents the first coordinated effort across these resources. Frameworks like Tripal and PlantGenIE focus on efficient deployment, web services for cross-talk, data visualization, and analytics to provide a robust environment for end users. As an example, the Elastic Search module developed by HWG allows one to search a gene, genome, marker, and other indexed objects in one database and locate results in other Tripal databases without executing independent searches on each website. Sharing development across a larger community allows forest tree databases to focus on the specific needs of their users. Their independent value exists in the additional curation, metadata acquisition, indexing, analytics, and visualization that is not delivered from the primary repositories. TreeGenes and Hardwood Genomics Web, are focused on expression data integration for non-model trees and metadata retrieval and cross-study analytics for population genetics studies. GDR is focused on improving access and visualization of genetic maps as well as breeding tools. The PlantGenIE framework is providing a robust platform for species with a reference genome, and advanced visualization for expression data that integrates across studies. All of these databases are also seeking stronger connections to more broadly plant focused repositories, such as Phytozome, PLAZA, and Planteome, that provide genetic and ontological resources that improve the utility of cross-site querying.

While tremendous advancements have been made through recent and focused development on these pivotal frameworks, several challenges remain for the forest tree community. As datasets become larger and more integrative, it is increasingly difficult for small database teams to keep up with the data capture and curation. With increasing access to reference genomes, large-scale population studies, and high throughput environmental data, biological databases must develop more efficient metadata capture, storage, and query capacity. These repositories will be tasked with implementing advanced natural language processing, automated metadata capture, and ontological term assignment to span not only genetic data, but associated phenotypic and environmental data. These latter categories encompass an expansive range, from traditional growth traits to canopy metrics, soil profiles, microbiomes, and metatranscriptomics. The biomedical community has paved the way for some of this technology but forest tree data, and the associated genetic resources, remain more heterogenous (Koleck et al., 2019). This heterogeneity is combined with high throughput technologies, such as remote sensing, that challenge existing cyberinfrastructure in terms of efficient transfer, storage, and query (Côté et al., 2018). Capturing data for large forest tree populations may involve storing millions of genotypes across thousands of individuals or hundreds of pangenomes. It will also rely on a combination of sequencing and phenotyping technologies that continue to evolve (Bolger et al., 2019). After the storage and minimal reporting requirements are established, the frameworks the databases are built upon will need to assist users in determining the most appropriate analytics and provide the required formatting for the queried data. While progress has been made in connecting data to workflows on high performance computing, such as Galaxy; systems that can recommend appropriate workflows are still in progress. The future of biological databases for all plants is reproducible workflows that represent the metadata associated with the original studies. Concerted efforts in this area and integration of new data types evolving from high throughput technologies will be key to advancing discovery for the forest tree community.

## Author Contributions

JW, MS, NS, DM, and SF designed the databases and software described. EG, NH, SB, TF, SZ, RR, PR, and LS developed the core TreeGenes and TreeGenes Tripal modules. MS, BC, AA, and MC developed the core HWG and HWG Tripal modules. NS and CM developed the core PlantGenIE. SJ developed the core GDR and GDR Tripal modules. SF developed the core Tripal. JW, MS, NS, and DM wrote the manuscript. All authors read and approved the manuscript.

## Conflict of Interest Statement

The authors declare that the research was conducted in the absence of any commercial or financial relationships that could be construed as a potential conflict of interest.

## Footnotes

1 http://www.treegenesdb.org

2 https://www.rosaceae.org

3 https://github.com/PlantGenIE/GenIECMS
